# Supplementary material for: AI-designed NMR spectroscopy RF pulses for fast acquisition at high and ultra-high magnetic fields
Source: Nat Commun. 2023 Jul 12;14:4144. doi: 10.1038/s41467-023-39581-4 (PMC10338431; doi:10.1038/s41467-023-39581-4)
Supplement: Supplementary file 1 — Supplementary Information [file 41467_2023_39581_MOESM1_ESM.pdf]

# Supplementary Information

# AI-designed NMR spectroscopy RF pulses for fast acquisition at high and ultra-high magnetic fields

Manu V.S.,[1] Cristina Olivieri,[1,†] and Gianluigi Veglia[a,*]

CONTENT:

**Supplementary Table 1:** New pulses generated by GENETICS-AI.

**Supplementary Fig. 1.** Amplitude-offset responses of GENETICS-AI pulses used in the RAPID-TROSY pulse sequence.

**Supplementary Fig. 2.** Amplitude-offset responses of UARev1 with a trailing delay of 0.75 times of pulse length.

**Supplementary Fig. 3.** Offset response for the binary PC9 (Bruker name PC9_4_90.1000) and REBURP pulses.

**Supplementary Fig. 4**. Effects of longitudinal and transverse relaxation ($R_1$ and $R_2$) on the GENETICS-AI excitation pulses.

**Supplementary Fig. 5.** Effects of longitudinal and transverse relaxation ($R_1$ and $R_2$) for the GENETICS-AI refocusing pulses.

**Supplementary Fig. 6.** Performance of the different TROSY-HSQC experiments for Maltose binding Protein (MBP) at various interscan delays.

**Supplementary Fig. 7**. Performance of the various TROSY-HSQC experiments for the RIIβ dimer at different interscan delays.

**Supplementary Table 1: New pulses generated by GENETICS-AI. Note that the nomenclature reflects the specific spin operation performed.**

| Pulse Name | Operation Type | Amplitude ($B_1$) | Total pulse Duration | Bandwidth | Reference |
|---|---|---|---|---|---|
| ***UA90ev1*** | Band Selective 90° on amide with J evolution | 5 kHz | 27.125*50 μs = 1356.25 μs | 0.9*$B_1$ | Figure 1 |
| ***URev1*** | Broadband 180° with J evolution | 16.667 kHz | 59*15 μs = 885 μs | 0.5*$B_1$ | Figure 1 |
| ***URev2*** | Broadband 180° with J evolution | 7.14 kHz | 22*35 μs = 770 μs | 0.55*$B_1$ | Figure 1 |
| ***UARev1*** | Band Selective 180° on Amide with J evolution | 5 kHz | 37.248*50 μs = 1862.4 μs | 1*$B_1$ | Figure 1 |
| ***UR1*** | Universal refocusing pulse | 7.1429 kHz | 7.093*35 μs = 248.255 μs | 0.8*$B_1$ | Figure 2 (DOI: 10.1039/D2CP01744J) |

U = Universal Rotation

A = Band selective pulse on amide resonances

R = Refocusing

ev = Chemical shift and J coupling evolution

The number at the end of the pulse name is an ID for indexing.

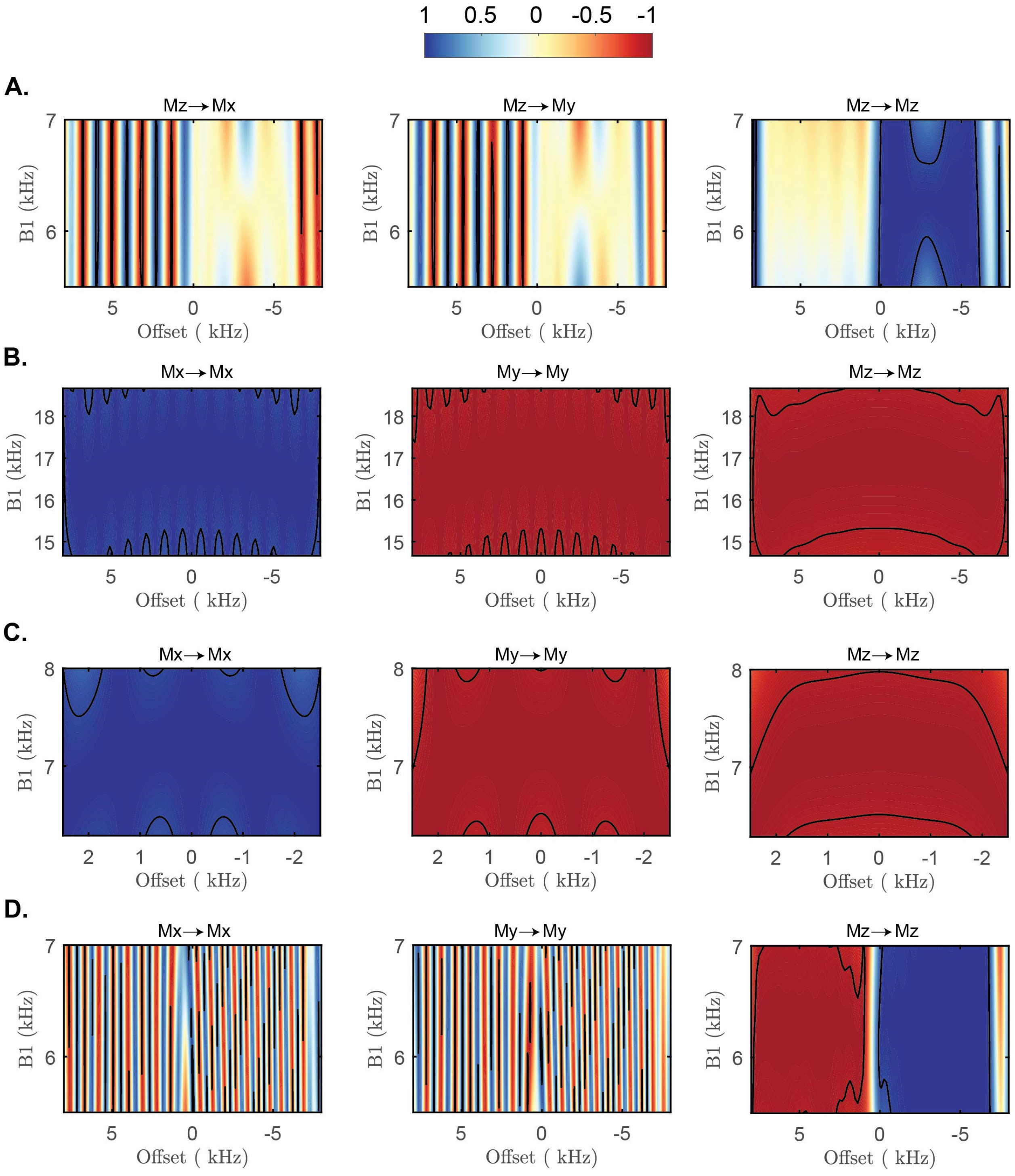


**Supplementary Fig. 1. Amplitude-offset responses of the GENETICS-AI generated pulses for the different components of the magnetization ($M_z$, $M_x$, and $M_y$).** A. UA90ev1, B. URev1, C. URev2, and D. UARev1. The spin operations and the characteristic of these pulses are reported in Table S1.

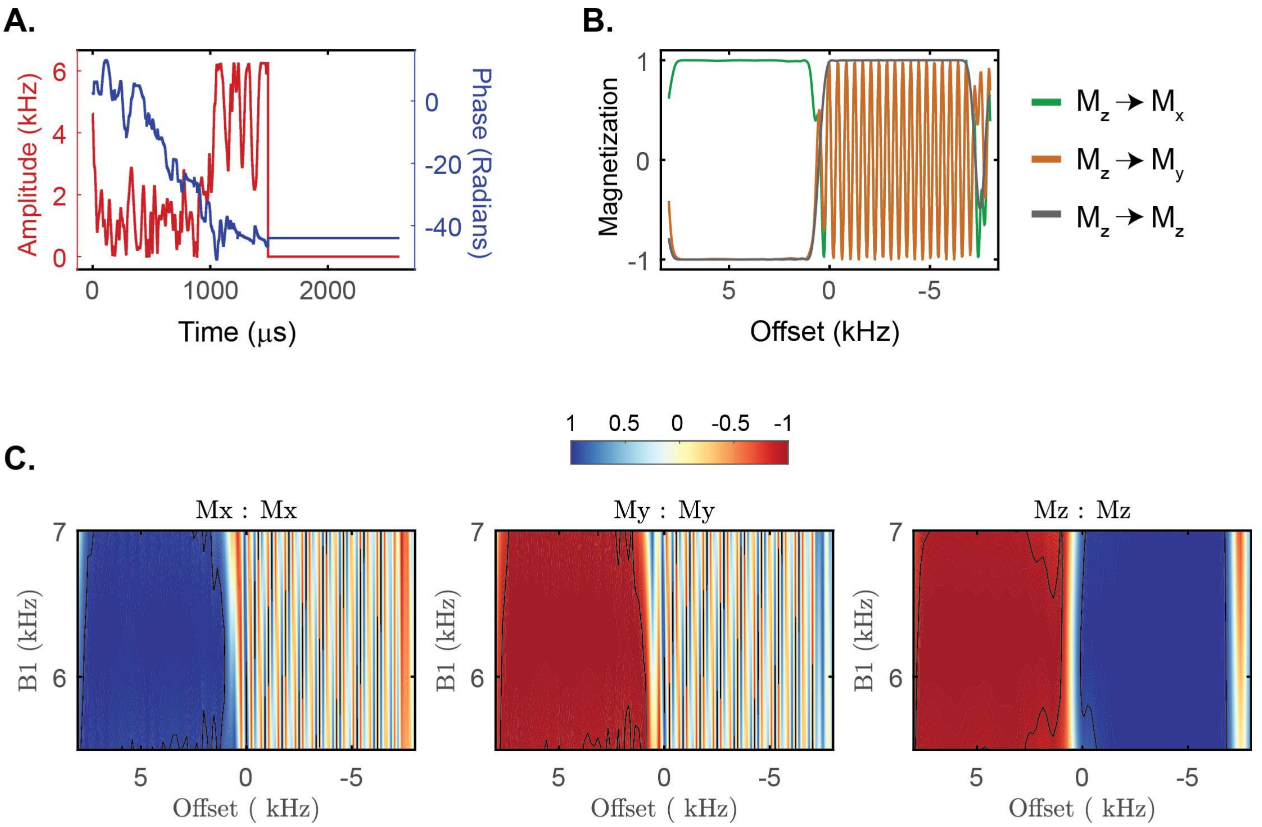


**Supplementary Fig. 2. Amplitude-offset responses of UARev1 with a trailing delay of 0.75 times the pulse length.** A. Pulse amplitude and phase vs. duration. B. Offset response of the magnetization components. C. Amplitude-offset responses of the magnetization components to UARev1 pulse. The details of these pulses are described in Supplementary Table 1.

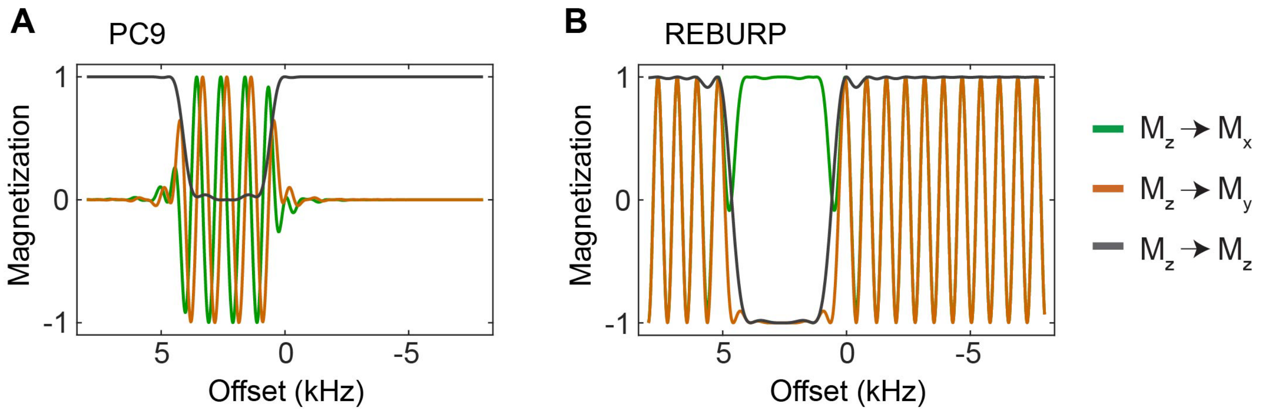


**Supplementary Fig. 3. Offset response for the binary PC9_90 (PC9_4_90.1000) and REBURP pulses.** A. PC9-90 with flip angle 90°. The pulse length was set to 2 ms and $B_1$ = 1 kHz. B. The REBURP pulse length was 1.35 ms and $B_1$ = 4.64 kHz.

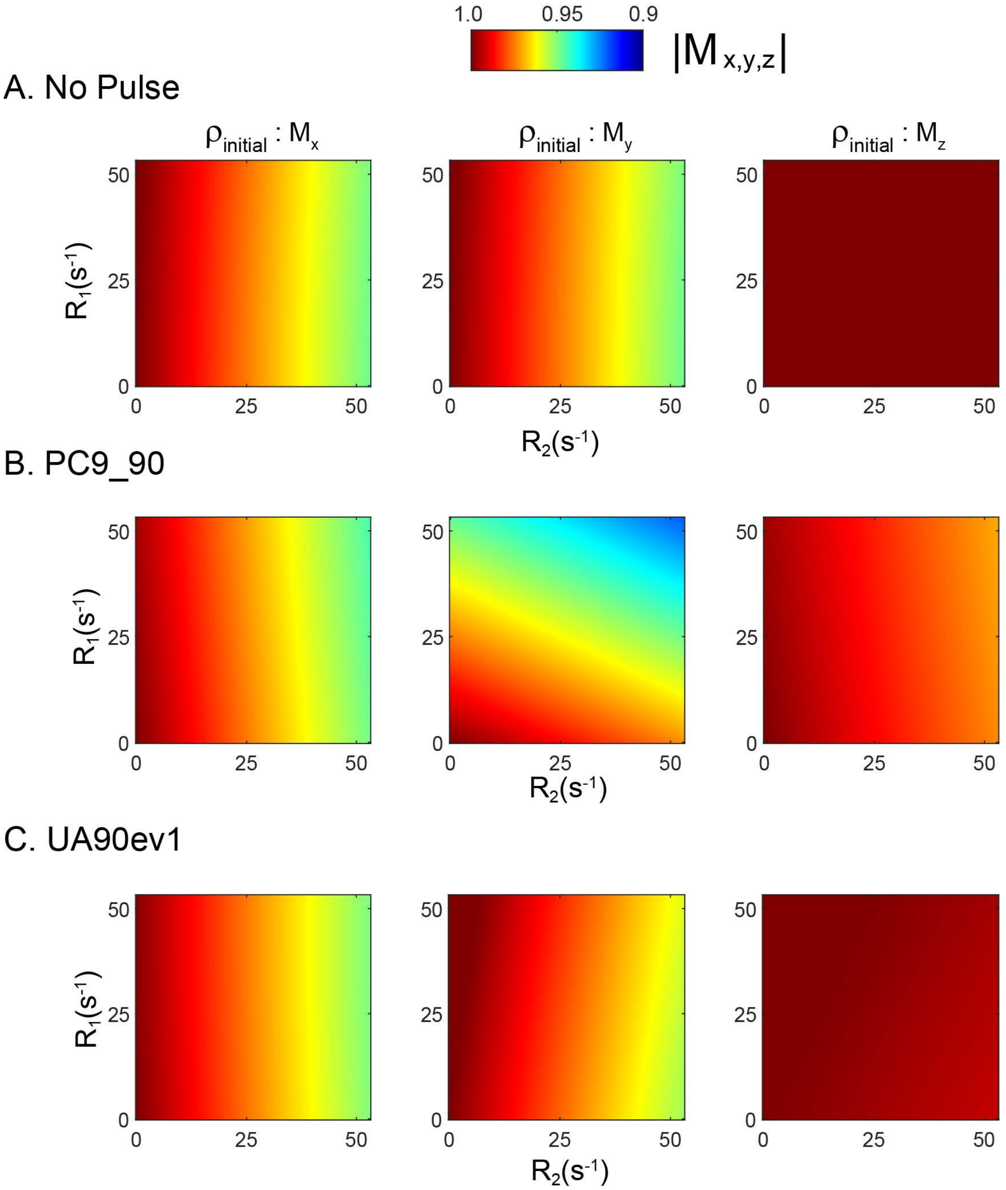


**Supplementary Fig. 4. Effects of longitudinal and transverse relaxation ($R_1$ and $R_2$) on the GENETICS-AI excitation pulses**. The 2D plots show the magnitude of the magnetization vector ($|Mxyz| = \sqrt{Mx^2 + My^2 + Mz^2}$) for different initial states ($\rho_{initial}$) of $M_x$ (left), $M_y$ (middle), and $M_z$ (right) for pulse lengths of 1 ms. (A) Reference state with no pulse is applied. (B) Effects on PC9_90 pulse. (C): Effects on the UA90ev1 pulse. The response for the PC9_90 pulse was measured on resonance (offset = 0 Hz). For the UA90ev1 pulse, the magnetization was monitored for an offset of 5 kHz.

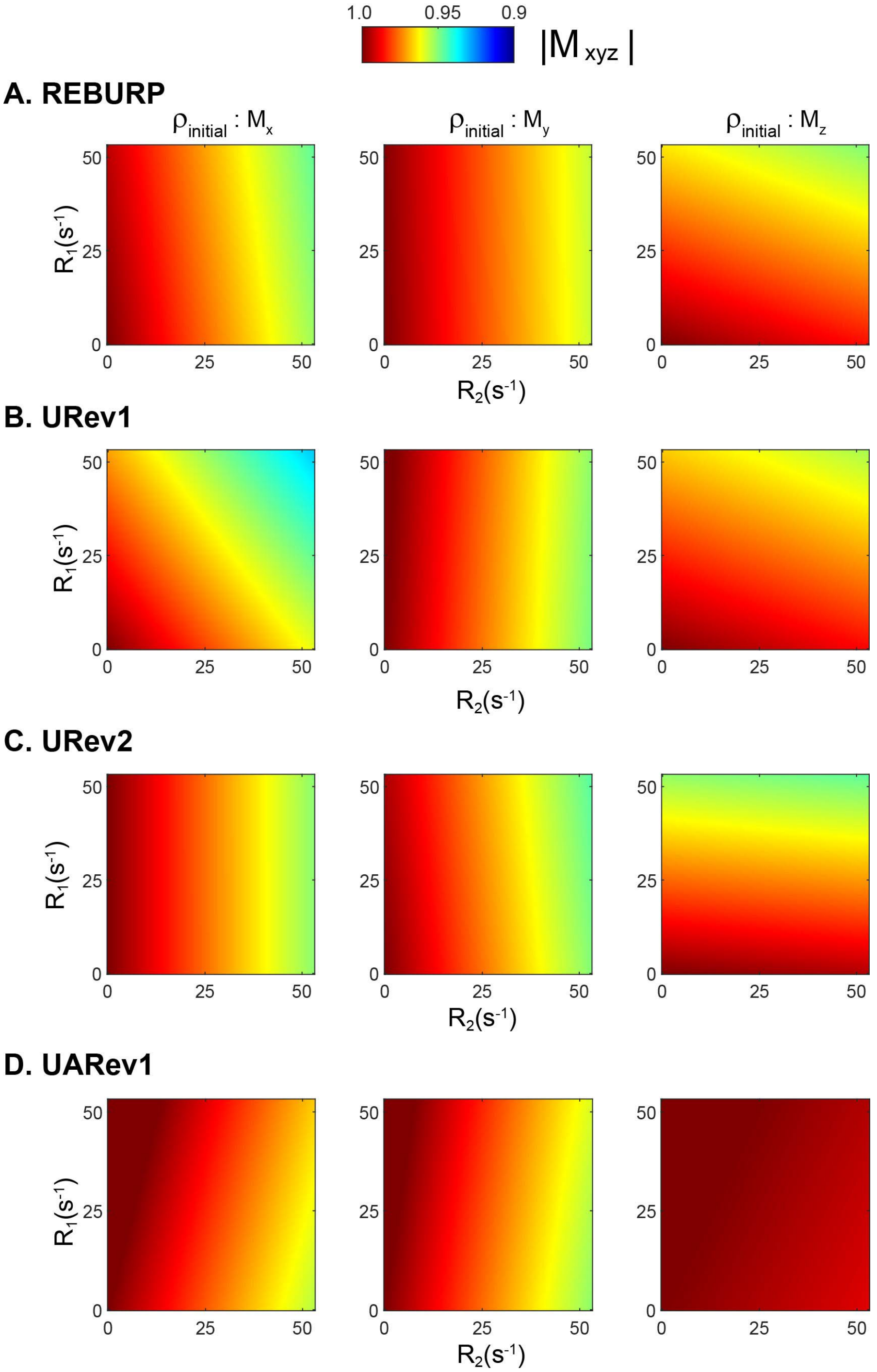


**Supplementary Fig. 5. Effects of longitudinal and transverse relaxation (*$R_1$* and *$R_2$*) on the GENETICS-AI refocusing pulses**. The 2D plots show the magnitude of the magnetization vector (|Mxyz| = $\sqrt{Mx^2 + My^2 + Mz^2}$) for different initial states ($\rho_{initial}$) of $M_x$ (left), $M_y$ (middle), and $M_z$ (right) for pulse lengths of 1 ms. (A) REBURP, (B) URev1, (C) URev2, and (D) UARev1. The response was measured by setting the offset on resonance for all the pulses, except for the UARev1 pulse, for which the magnetization was monitored by setting the offset at 5 kHz.

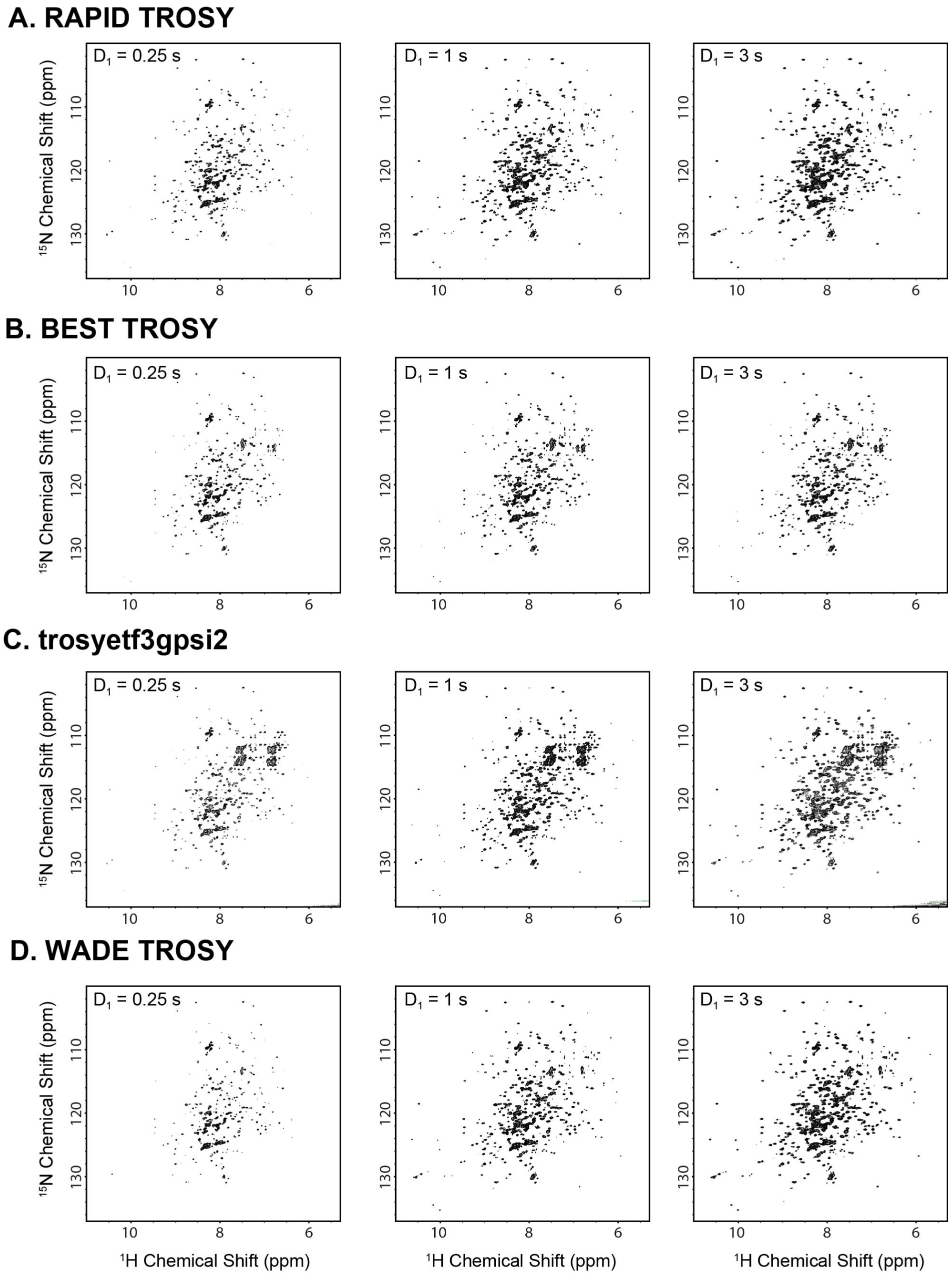


**Supplementary Fig. 6. Performance of the different TROSY-HSQC experiments for Maltose binding Protein (MBP) at different interscan delays.** (A) RAPID-TROSY, (B) BEST-TROSY, (C) trosyetf3gpsi2, and (D) WADE-TROSY. All the experiments are performed in a Bruker 900 MHz Avance III spectrometer with 5-mm TCI CryoProbe. We have used the same acquisition and processing parameters for all the experiments. 160 and 1536 complex fid points were acquired in indirect and direct dimensions, respectively, with 4 scans and 16 dummy scans at temperature 300 K. All the spectra were processed using nmrPipe. We have used sine-bell apodization (SP) with an offset of 0.4 in both direct and indirect dimensions. Before Fourier transform, the complex fid matrix was zero-filled to a final size of 256x1536.

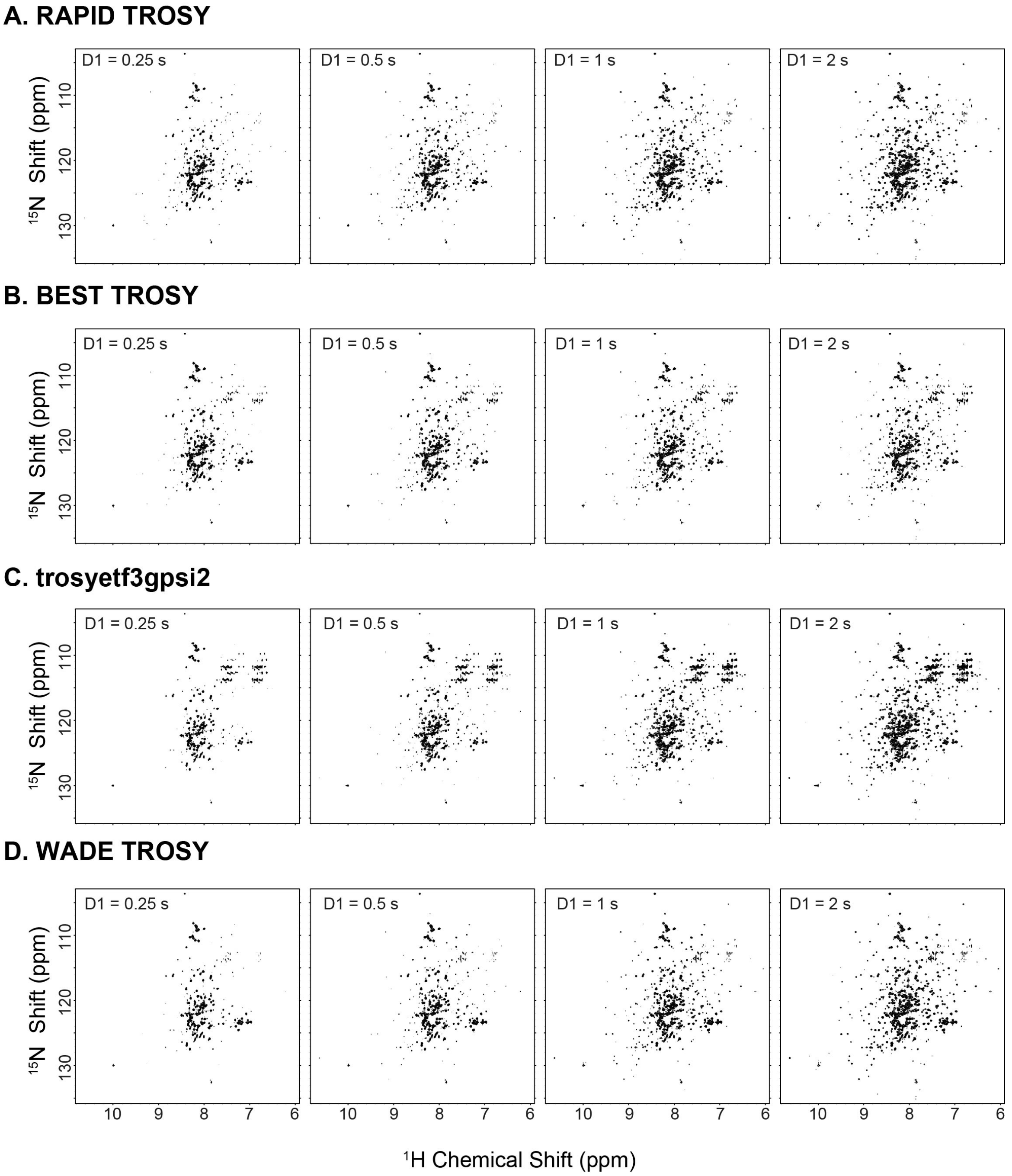


**Supplementary Fig. 7. Performance of the various TROSY-HSQC experiments for the RIIβ dimer at different interscan delays.** (A) RAPID-TROSY, (B) BEST-TROSY, (C) trosy-etf3gpsi2, and (D) WADE-TROSY. All the experiments are performed in a Bruker 900 MHz Avance III spectrometer with 5-mm TCI cryoprobe. We have used the same acquisition and processing parameters for all the experiments. 128 and 1536 complex fid points were acquired in indirect and direct dimension, respectively, with 32 scans and 32 dummy scans at temperature 300 K. All the spectra were processed using nmrPipe. We have used sine-bell apodization (SP) with an offset of 0.4 in both direct and indirect dimensions. Before Fourier transform, the complex fid matrix was zero-filled to a final size of 256x1536.
